# Iso-seq and RNA-seq data from ML-2 acute myeloid leukemia cells overexpressing the ZCCHC10 gene

**DOI:** 10.1016/j.dib.2025.112048

**Published:** 2025-09-05

**Authors:** Hao Zhou, Jianlin Zhou, Yichong Ning

**Affiliations:** a College of Clinical Laboratory, Changsha Medical University, Changsha 410219, Hunan, China; b College of Life Science, Hunan Normal University, Changsha 410081, Hunan, China; c Chongzuo Key Laboratory of Biomedical Clinical Transformation, The People’s Hospital of Chongzuo, Youjiang Medical University for Nationalities, Chongzuo 532200, Guangxi, China

**Keywords:** RNA sequencing, Transcriptomics, Alternative processing, Alternative splicing

## Abstract

ZCCHC10 (zinc finger CCHC-type containing 10) has been shown to play a tumor suppressive role in acute myeloid leukemia (AML), but the underlying mechanism is not clear. Many ZCCHC proteins are involved in RNA metabolism, such as synthesis, splicing and stability of RNA. To investigate the role of ZCCHC10 in RNA metabolism, we performed PacBio single-molecule long-read isoform sequencing (Iso-seq) and Illumina short-read RNA sequencing (RNA-seq) on ML-2 cells stably overexpressing ZCCHC10 (designated ML2T) and stably overexpressing an empty vector (designated ML2C). The Iso-seq data consists of full-length transcripts from ML2T and ML2C and can be used by researchers to identify mRNA isoforms and decipher the events of alternative processing, including alternative transcription initiation, alternative splicing, and alternative polyadenylation. The RNA-seq data provides the transcript profiles from ML2T and ML2C and can be used by researchers to study the effects of overexpression of ZCCHC10 on gene expression. Integrated analysis of RNA-seq and Iso-seq datasets can reveal the impact of ZCCHC10 overexpression on alternative processing in AML.

Specifications table

**Table:** 

Subject	Health Sciences, Medical Sciences & Pharmacology
Specific subject area	Cancer research, Transcriptomics, Alternative processing
Type of data	Table, Figure, Raw, Processed
Data collection	Total RNA was extracted from ML-2 cells stably overexpressing ZCCHC10 (referred to as ML2T) and from cells stably overexpressing the empty vector (referred to as ML2C) using TRIzol reagent (Invitrogen, Carlsbad, CA, USA), and mRNA was subsequently enriched with oligo(dT) beads. For RNA-seq, libraries were prepared using the NEBNext Ultra RNA Library Prep Kit (NEB, Ipswich, MA, USA) and sequenced using the paired-end 150 bp read strategy on the Illumina NovaSeq 6000 System (San Diego, CA, USA). For Iso-Seq, libraries were prepared using the SMRTbell Prep Kit (PacBio, San Diego, CA, USA) and sequenced using the PacBio Sequel II.
Data source location	Hunan Normal University, 36 Lushan Road, Changsha, China
Data accessibility	Repository name: NCBI BioProject Data identification number: PRJNA667353, PRJNA1002811 Direct URL to data: https://www.ncbi.nlm.nih.gov/geo/query/acc.cgi?acc=GSE159016 https://www.ncbi.nlm.nih.gov/sra/SRX9243084 https://www.ncbi.nlm.nih.gov/sra/SRX9243085 https://www.ncbi.nlm.nih.gov/sra/SRX9243086 https://www.ncbi.nlm.nih.gov/sra/SRX9243087 https://www.ncbi.nlm.nih.gov/sra/SRX9243088 https://www.ncbi.nlm.nih.gov/sra/SRX9243089 https://www.ncbi.nlm.nih.gov/sra/SRX21274348 https://www.ncbi.nlm.nih.gov/sra/SRX21274349
Related research article	None

## 1 Value of the Data

• The RNA-seq datasets can be used by researchers to study the effects of ZCCHC10 overexpression on gene expression.
• The Iso-seq datasets can be used by researchers to identify mRNA isoforms and decipher the events of alternative processing, including alternative transcription initiation, alternative splicing, and alternative polyadenylation.
• Integrated analysis of RNA-Seq and Iso-seq datasets may reveal the effects of ZCCHC10 overexpression on alternative processing in AML.

## 2 Background

Our previous study has shown that ZCCHC10 (zinc finger CCHC-type containing 10) plays a tumor suppressive role in AML [1]. ZCCHC10 belongs to the zinc finger CCHC-type (ZCCHC) family of proteins, all of which contain conserved C-X2-C-X4-H-X4-C sequences (where C is cysteine, H is histidine and X is any amino acid)[2,3]. It is generally believed that ZCCHC motifs have the ability to bind RNA [2,3]. A total of 25 human ZCCHC genes are annotated in the GenBank database. Most of them play a role in RNA metabolism, including synthesis, splicing, stability, N6-methyladenosine modification and translation of RNA [[3], [4], [5]]. Therefore, we hypothesize that ZCCHC10 may also be involved in RNA metabolism. To investigate the role of ZCCHC10 in RNA metabolism, we performed PacBio single-molecule long-read isoform sequencing (Iso-Seq) and Illumina short-read RNA sequencing (RNA-seq) to comprehensively examine the changes in the transcriptome following overexpression of the ZCCHC10 gene in the ML2 cell line. RNA-seq data can be used to analyze mRNA expression levels and mRNA isoforms. However, RNA-seq usually generates reads of 50–200 bp, which must be assembled into complete transcripts using bioinformatics tools. Assembly errors complicate the accurate characterization of isoforms. Iso-seq directly sequences the cDNA of a complete transcript isoform from the 5′ end to the 3′ polyA tail. Therefore, Iso-seq data are often used to validate transcripts identified by RNA-seq, and integrated analysis of RNA-seq and Iso-seq data can accurately identify alternative processing.

## 3 Data Description

We present Iso-seq and RNA-seq data from ML2T and ML-2 cells.

The raw Iso-seq data have been deposited in the Sequence Read Archive (SRA) database of the NCBI BioProject under accession number PRJNA1002811. The SRA data accession numbers for two samples are SRX21274348 and SRX21274348. The polymerase reads, subreads and circular consensus sequence (CCS) are summarized in Table 1. A total of 83,836 and 82,654 isoforms were identified in ML2C and ML2T cells, respectively (Table 2).

**Table 1:** Summary of Iso-seq data.

Sample	Polymerase_reads		subreads		CCS	
	number	Average length	number	Average length	number	Average length
ML2C	1361,544	106,840.44	49,262,167	2908.58	1361,544	3398.52
ML2T	1521,028	106,654.21	55,568,208	2875.02	1521,028	3331.22

**Table 2:** Summary of the isoforms in Iso-seq data.

Sample	ML2C	ML2T
Total number of isoforms	83,836	82,654
Number of isoforms of known genes	20,646	21,246
Number of novel isoforms of known genes	61,789	60,520
Number of isoforms of unknown genes	1404	891

The raw RNA-seq data have been deposited in the SRA database of NCBI BioProject under accession number PRJNA667353. The SRA data accession numbers for six samples are SRX9243084, SRX92430845, SRX9243086, SRX9243087, SRX92430848, and SRX9243089. The processed data are stored in the GEO database under accession number GSE159016. The raw and cleaned data are summarized in Table 3. The clead reads mapped to the Homo sapiens GRCh38.87 genome are summarized in Table 4.

**Table 3:** Summary of RNA-Seq data.

Sample	ML2C1	ML2C2	ML2C3	ML2T1	ML2T2	ML2T3
Raw reads number	46,452,748	45,855,248	43,824,936	41,057,552	45,264,082	48,770,324
Raw bases number	6967,912,200	6878,287,200	6573,740,400	6158,632,800	6789,612,300	7315,548,600
Clean reads number	45,037,410	44,278,562	42,347,324	40,013,802	44,159,620	47,628,076
Clean reads rate (%)	96.95	96.56	96.63	97.46	97.56	97.66
Clean bases number	6755,611,500	6641,784,300	6352,098,600	6002,070,300	6623,943,000	7144,211,400
Raw Q30 bases rate (%)	93.8	93.47	93.26	93.52	93.46	93.45
Clean Q30 bases rate (%)	94.12	93.8	93.69	93.85	93.77	93.8

* Q30: Percentage of bases with quality score (Qphred) > 30.

**Table 4:** Summary of reads mapping to reference genome in RNA-seq data.

Sample	Total reads	Mapped reads	Mapping rate	Total gene number	Group
ML2C1	45,037,410	43,828,596	0.9732	24,593	ML2C
ML2C2	44,278,562	43,057,020	0.9724	24,481	ML2C
ML2C3	42,347,324	41,099,811	0.9705	24,142	ML2C
ML2T1	40,013,802	38,870,228	0.9714	25,033	ML2T
ML2T2	44,159,620	42,881,223	0.9711	25,233	ML2T
ML2T3	47,628,076	46,222,507	0.9705	25,624	ML2T

## 4 Experimental Design, Materials and Methods

### 4.1 Sample preparation

ML-2 acute myeloid leukemia cells stably overexpressing ZCCHC10 or the empty vector were constructed [1]. Total RNA was extracted with TRIzol reagent. The purity of RNA was checked by spectrophotometer (Kaiao, Beijing, China) and RNA integrity was measured using the RNA Nano 6000 Assay Kit on the Bioanalyzer 2100 System (Agilent Technologies, Santa Clara, CA, USA).

### 4.2 PacBio isoseq

A total of 4 μg of mRNA was used to synthesize double-stranded cDNA using the SMARTer PCR cDNA Synthesis Kit (Takara Biotechnology, Dalian, China). Subsequently, 1 μg of cDNA was amplified using the KAPA HiFi PCR Kit (KAPA Biosystems, Cape Town, South Africa). Fragmentation screening was performed using the BluePippin system (Sage Science, Beverly, MA, USA). The SMRTbell library was prepared using the SMRTbell Template Prep Kit 1.0. Binding of the SMRTbell templates to the polymerases was measured using the Sequel II Binding Kit, followed by primer annealing. Sequencing was performed using the Pacific Bioscience Sequel II platform. A total of two SMRT cells were performed.

The original sequence that is read during sequencing is called a polymerase read. After removing the adapters, each insertion fragment generates a subread for each sequencing. Consensus sequences generated from subreads (more than one) derived from the same polymerase read are called CCS sequences. Isoseq3 (Pacbio) is used for clustering and error correction to obtain high-quality isoforms. The clustered high-quality transcript sequences were aligned to the Homo_sapiens.GRCh38.87 genome using GMAP software [6]. The alignment results were compared with the genome annotation file GTF using MatchAnnot software (Pacbio) to obtain all transcript sequences.

### 4.3 Illumina RNA-seq

RNA-seq libraries were prepared using the NEBNext Ultra RNA Library Prep Kit for Illumina (NEB, Ipswich, MA, USA) according to the manufacturer’s instructions. In brief, mRNA was purified from total RNA using oligo (dT)-bound magnetic beads and fragmented in NEBNext First Strand Synthesis Reaction Buffer. The generated short fragments of mRNA were used to synthesize the first strand of cDNA with random hexamer primer and RNase H. The second strand of cDNA was then synthesized with buffer, dNTPs, DNA polymerase I and RNase H. The double stranded cDNA fragments were purified using the QiaQuick PCR kits (Qiagen, Hilden, Germany), subjected to end repair and 3′ A-tailing, and ligated to the sequencing adapter. The fragments were purified and then enriched by PCR to obtain the final sequencing library.

The RNA concentration of the library was measured with the Qubit RNA Assay Kit in the Qubit 3.0 Fluorometer (Invitrogen, Carlsbad, CA, USA). The size of the inserts was determined using the Agilent Bioanalyzer 2100 system (Agilent Technologies, CA, USA). Libraries were sequenced on an Illumina NovaSeq 6000 (San Diego, CA, USA) and 150 bp paired-end reads were generated.

The raw image data were converted into sequenced reads (raw reads) by bcl2fastq software (Illumina) and saved as FASTQ (Fq) files. The sequenced reads were trimmed for adaptor sequence, and masked for low-complexity or low-quality sequence to obtain clean reads. The clean reads were mapped to the Homo_sapiens.GRCh38.87 genome using HISAT2 v2.1.0 [7]. FPKM (Fragments Per Kilobase Millon Mapped Reads) was calculated to estimate the expression level of genes in each sample [8].

## Limitations

None.

## Ethics Statement

The authors have read and follow the ethical requirements for publication in Data in Brief and confirming that the current work does not involve human subjects, animal experiments, or any data collected from social media platforms.

## Credit Author Statement

**Hao Zhou:** Investigation, Formal analysis; Writing- Original draft preparation, Writing- Reviewing and Editing. **Jianlin Zhou:** Supervision, Funding acquisition, Writing- Original draft preparation, Writing- Reviewing and Editing. **Yichong Ning:** Conceptualization, Funding acquisition, Writing- Original draft preparation, Writing- Reviewing and Editing

## Data Availability

SRARNA-seq data (Original data)
SRAIso-seq data (Original data)
